# Bioactivity of Essential Oils for Mitigation of *Listeria monocytogenes* Isolated from Fresh Retail Chicken Meat

**DOI:** 10.3390/foods10123006

**Published:** 2021-12-04

**Authors:** Alaa Eldin M. A. Morshdy, Mohammed S. Al-Mogbel, Mohamed E. M. Mohamed, Mohamed Tharwat Elabbasy, Azza K. Elshafee, Mohamed A. Hussein

**Affiliations:** 1 Food Control Department, Faculty of Veterinary Medicine, Zagazig University, Zagazig 44519, Egypt; A.Morshedy@zu.edu.eg (A.E.M.A.M.); mt.elabbasy@uoh.edu.sa (M.T.E.); elged2010@yahoo.com (M.A.H.); 2 Clinical Laboratory Sciences Department, College of Applied Medical Sciences, Ha’il University, Ha’il P.O. Box 2240, Saudi Arabia; msm_hhscb@hotmail.com; 3 Zoonoses Department, Faculty of Veterinary Medicine, Zagazig University, Zagazig 44519, Egypt; bishet68@yahoo.de; 4 Public Health Department, College of Public Health and Health Informatics, Ha’il University, Ha’il P.O. Box 2240, Saudi Arabia

**Keywords:** *Listeria monocytogenes*, antimicrobial agents, essential oils, multidrug-resistance, foodborne disease

## Abstract

*Listeria monocytogenes* is one of the most severe foodborne pathogens found in several habitats. Therefore, this study aims to investigate the antilisterial activity of different essential oils (EOs) against multidrug-resistant (MDR) *L. monocytogenes* strains isolated from fresh chicken meat. Our results showed that the prevalence of *L. monocytogenes* in the examined samples was 48%. Seventy-eight isolates were identified as *L. monocytogenes*. Out of these, 64.1% were categorized as MDR and were categorized in 18 patterns with 50 MDR isolates. One isolate was selected randomly from each pattern to investigate their biofilm-forming ability, resistance, and virulence genes incidence. Out of 18 MDR isolates, 88.9% showed biofilm-forming ability. Moreover, the most prevalent resistance genes were *erm*B (72%), *aad*A (67%), *pen*A (61%), and *flo*R genes (61%). However, the most prevalent virulence genes were *inl*A (94.4%), *prf*A (88.9%), *plc*B (83.3%), and *acta*A (83.3%). The antilisterial activity of EOs showed that cinnamon bark oil (CBO) was the most effective antilisterial agent. CBO activity could be attributed to the bioactivity of cinnamaldehyde which effects cell viability by increasing the bacterial cell electrical conductivity, ion leakage, and salt tolerance capacity loss. Therefore, CBO could be an effective alternative natural agent for food safety applications.

## 1. Introduction

Despite modern advances in food processing and preservation techniques, food protection is a growing public health concern [1]. In particular, the survival of microorganisms in foods is a critical concern, as it can cause food spoilage, infection, and illness [2]. *Listeria monocytogenes* is considered one of the primary foodborne pathogens because of its widespread prevalence and high tolerance against severe environmental conditions such as salt, temperature, and pH, among all known *Listeria* spp. [3]. *L. monocytogenes* is a foodborne pathogen that can be found in various food products and is considered a significant concern for foodborne bacterial pathogens [4]. Clinical disease caused by *L. monocytogenes* infections varies from gastroenteritis with fever to invasive infections that require hospitalization and may result in death [1].

*L. monocytogenes*, the main cause of listeriosis, is an intracellular bacterium that can invade several cell types. In addition, *L. monocytogenes* can cross the intestinal and cross barriers between the blood vessels of the brain and placenta in the case of pregnancy and even could affect the fetus [5]. Human listeriosis is a foodborne disease associated with the ingestion of infected food products, with a mortality rate reaching up to 20% [6].

Different preservation methods (chemical preservatives, refrigeration, and heating) have been commonly used throughout food production to control foodborne pathogens. On the other hand, incomplete inactivation of pathogenic agents was widely documented, and post-treatment contamination may reintroduce pathogenic agents into food products [7,8,9]. Furthermore, consumers’ fear is increasing concerning the use of chemical preservatives in the food industry due to their possible side effects and toxicity [10]. Therefore, tremendous effort has been expended in creating alternative natural additives for improving the quality of food products, in particular the microbiological quality.

Plant essential oils (EOs) and extracts consider a natural antimicrobial source in the foods industry as preservation and flavoring agents [7,8,9,10,11,12]. Plant extracts are generally healthy according to the modern extensive toxicological studies and due to a lack of known adverse impact during historical use [10]. Plant extracts contain a high concentration of phytochemical compounds and secondary metabolites that inhibit pathogenic agents [13]. Plant extracts have been proven to have wide antimicrobial efficacy toward foodborne pathogenic bacteria such as *E. coli*, *Listeria innocua* [14], and *L. monocytogenes* [15,16]. Natural food additives are commonly used, such as Proallium (garlic and onion extracts) [17] and Cycrom (20% citrus extract and organic acids) [18], which have already been commercialized. However, it is estimated that only 10% of the world’s 250,000–500,000 plant species have been assessed, and few studies have investigated the chemical composition and the mode of action of plant extracts [19]. Therefore, it is important to investigate the activity of different plant extracts and essential oils as antimicrobial agents against food products spoilage pathogens such as *L. monocytogenes* to indicate their potential in food safety applications.

Despite the importance of chicken meat in the Egyptian market, few studies have been conducted to study *L. monocytogenes* in fresh marketed chicken meat in Zagazig City, Egypt. As well, to the best of our knowledge, this study is the first to explore antibiotic-resistant bacterial strains in these markets and at the same time investigate the effect of traditional oils on these bacterial strains. Therefore, this study aims to achieve the following goals:

1. Investigate the prevalence of *L. monocytogenes* in fresh retail chicken meat;
2. Detect the antibiogram profile of the isolated *L. monocytogenes*;
3. Evaluate the biofilm-forming ability (BFA), virulence, and resistance genes;
4. Investigate the activity of different EOs as antimicrobial agents against multidrug-resistant *L. monocytogenes*;
5. Study the effect of EOs on bacterial cell viability and integrity loss, as indicated by increased electrical conductivity, ion leakage, and salt tolerance capacity loss.

The results obtained in this study are expected to reveal valuable details about the bioactivity of EOs as antilisterial agents and expand our understanding of the antibacterial mechanism. Thus, this study could contribute to food preservative and pharmaceutical industries applications.

## 2. Materials and Methods

### 2.1. Sampling

**Figure 1:**
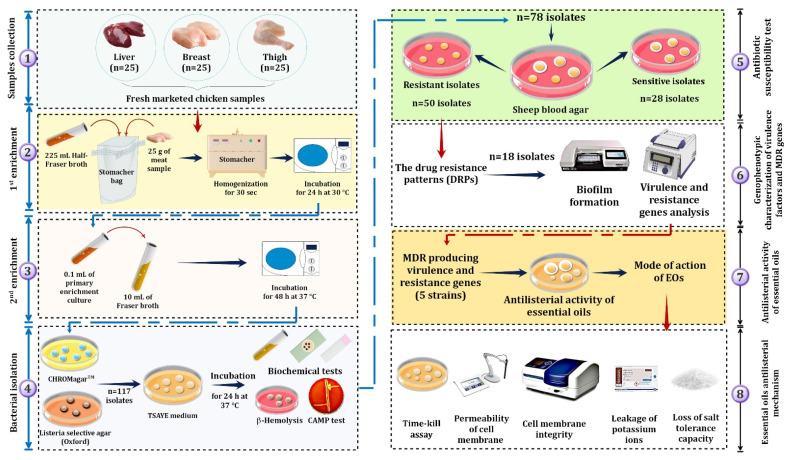
Schematic diagram of the experimental setup design for screening and characterizing *L. monocytogenes* isolated from fresh retail chicken meat samples.

As illustrated in the experimental setup, Figure 1, seventy-five fresh retail chicken meat samples, including breasts, thighs, and livers, were used in this study and randomly collected from 12 different supermarkets and four different brands in ElSharkia, Egypt, from April to November 2020. Afterward, to avoid cross-contamination, each sample was labeled and delivered in a sterile plastic storing bag and transferred in a cooling icebox (4 °C) immediately to the hygienic food laboratory, Faculty of Veterinary Medicine, ElSharkia University, within three hours after sampling and immediately analyzed.

### 2.2. Isolation of L. monocytogenes

All collected samples were examined for *L. monocytogenes* incidence based on the International Standards Organization guidelines [20]. As shown in Figure 1, the primary enrichment and secondary enrichment were performed for chicken meat samples. Afterward, for the bacterial isolation, a loopful of enrichment cultures was streaked on Listeria Selective Agar (Oxoid Hampshire, UK) supplemented with SR0140E (Oxford formulation) and CHROMagarTM Listeria (CHROMagar, Paris, France).

On CHROMagar^TM^, blue colonies surrounded by a white halo were isolated, while on Oxford agar, brown colonies surrounded by a black halo were isolated. To purify the colonies, they were streaked on the surface of trypticase soy agar supplemented with 0.6% yeast extract (TSAYE) and incubated for 24 h at 37 °C. Gram staining, oxidase, catalase, motility, spore formation, acid production from mannitol, D-Xylose, and L-Rhamnose tests were used to confirm the colonies from TSAYE as *L. monocytogenes*. Additionally, *β*-hemolytic activity and Christine–Atkins–Munch-Petersen (CAMP) were performed based on Bergey’s Manual of Systematic Bacteriology [21]. A stock culture of *L*. *monocytogenes* serotype 1/2b and *Staphylococcus aureus* ATCC 25923 were obtained from the Food Control Department, Zagazig University [22] and used as reference strains for biochemical tests and the CAMP test, respectively.

### 2.3. Antibiotics Susceptibility Test

In order to detect MDR *L. monocytogenes* isolates, fresh bacterial colonies of *L. monocytogenes* isolates were separately grown at 37 °C in brain heart infusion broth (BHI; Merck, Darmstadt, Germany) for 24 h, and each inoculum was cultured on Mueller Hinton Agar (MHA) with 5% sheep blood (Merck, Darmstadt, Germany). Susceptibility to 14 different antibiotics (MAST, Merseyside, UK) is listed in Table 1. The antibiotic selection was performed based on its effectiveness against Gram-positive bacteria. The antibiotics susceptibility test (AST) was determined using the standard disk diffusion Kirby–Bauer [23]. The inhibition zone diameters (IZD) were measured, including the disc diameter, the results were interpreted as resistant, intermediate, or susceptible, according to Clinical and Laboratory Standards Institute [24] for *S. aureus* ATCC 25923 because of the lack of specific standards for *L. monocytogenes* [25] (Table S1). The multiple antibiotic resistance (MAR) index was calculated according to Osundiya et al. [26].

### 2.4. Molecular Identification, Resistance, and Virulence Genes Detection

The selected isolates based on AST results were grown on Tryptone Soy Yeast Extract Agar (TSYEA) to extract the bacterial DNA and perform PCR assay as described by Osman et al. [27]. The genus was confirmed by PCR assay using specific primers for *L. monocytogenes* 16S rRNA gene (Supplementary Materials, Table S2) [28,29,30,31,32,33,34,35,36]. Amplification conditions were denatured (94 °C for 4 min), then followed by 25 amplification cycles (60 s at 94 °C, 60 °C for 60 s, and 72 °C for 60 s) with a final extension (5 min at 72 °C). *Escherichia coli* strain (ATCC 25922) and *L. monocytogenes* strain (ATCC 19115) were used as negative and positive control, respectively. The presence of Listeria pathogenicity island (LIPI-1) virulence genes (*plc*A, *plc*B, *prf*A, and *act*A), an adhesion protein (*lap)*, internalin proteins encoding genes (*inl*A, *inl*B, *inlC*, and *inl*J), Listeriolysin O gene (*hly*A), and a flagellin protein (*fla*A) were detected. In addition, the presence of resistance-encoding genes, namely, aminoglycoside adenyltransferase (*aad*A), *β*-lactamase–ampicillin resistance gene (*amp*C), vanillate o-demethylase oxygenase subunit (*van*A and *van*B); erythromycin resistance genes (*ere*A, *ere*B, and *erm*B); penicillin-binding protein gene (*pen*A), florfenicol export protein (*flo*R), chloramphenicol transporter nonenzymatic chloramphenicol-resistance protein (*cml*A), the tetracycline resistance genes (*tet*A and *tet*B) were detected using primers that are listed in the Supplementary Materials, Table S3 [37,38,39,40,41,42,43], and the PCR assay was performed as described by Osman et al. [27]. The PCR assay was performed on the selected isolates and reference strains. The resulting DNA sequence data were compared to data in the GenBank database using the BLAST algorithm available at the National Center for Biotechnology Information website. The phylogenetic analysis was performed using Mega 6 software. The phylogenetic tree analysis was constructed using the maximum-likelihood method.

### 2.5. Biofilm Quantification

The microtiter plate assay (MPA) was carried out according to Lee et al. [44] with minor modifications. In brief, selected overnight growing colonies were diluted to obtain an OD_600_ of 0.1 in each growth medium. A volume of 200 μL of the bacterial solution was transferred to 96-well microplates in triplicate wells. As a negative control, a sterile medium was used. Microplates were incubated for 24 h in static conditions at 30 °C. Plates were placed upside down, and then planktonic cells and the media were removed by tapping gently. Wells were washed using sterile saline solution (300 μL; 8.5 g NaCl/L) to remove loosely attached bacteria. Afterward, biofilms were fixed with 300 μL of ethanol (96% *v*/*v*) for 20 min then dried at 25 °C after ethanol removal.

For bacterial staining, 220 μL/well of crystal violet (0.1% *w*/*v*; CV; Merck, Darmstadt, Germany) was added, and plates were incubated in static conditions for 30 min. The solution was then removed by sharply placing the plates upside down. Wells were washed three times with 300 μL of saline solution and dried at 25 °C, then filled with acetic acid (150 μL; 33% *v*/*v*). Plates were set on a plate shaker for 10 min with slight agitation, and the amount of destained CV was assessed by reading OD_600_ using a microplate reader (BioTek EL800; Winooski, VT, USA). The experiment was performed in triplicates.

### 2.6. Gas Chromatography/Mass Spectrometry (GC/MS) Analysis of EOs

Seven EOs, namely, cinnamon bark oil (CBO), thyme (wild) oil (TWO), thyme (red) oil (TRO), thyme (geraniol) oil (TGO), coriander oil (CRO), lavender (true) oil (LTO), and rosemary oil (RO), were purchased from National Research Center (NRC Cairo, Egypt) based on their antilisterial activity in a literature survey and their use in traditional medicine [7,8,9,10,11,12,15] to evaluate their antilisterial activity against MDR *L. monocytogenes*. EOs were stored at 4 °C for further experiments. EOs chemical composition analysis was assessed using the Agilent 6890 N gas chromatograph (Agilent Technologies; Palo Alto, CA, USA) equipped with an HP-5MS capillary column (30 m × 0.25 mm × 0.25 µm). The oven temperature was adjusted to increase from 60 to 250 °C at a rate of 4 °C per min for 15 min. Transfer line temperature was 250 °C. Helium was used as the carrier gas with a flow rate of 1 mL/min. The EO sample was diluted in pentane, and the injection volume was 1 μL, while the split/splitless injector temperature was set at 280 °C. The EO sample was injected in the split mode with a split ratio of 40.8/1. However, a quadrupole mass spectrometer was scanned over the 35–465 *m*/*z* with an ionizing voltage of 70 eV and an ionization current of 150 mA. MS ion source and MS quadrupole temperatures were 230 °C and 150 °C, respectively. EOs compositions were identified by comparison of their mass spectra (MS) and retention indices (RI) the Wiley Registry of Mass Spectral Data, 6th Edition (Wiley Interscience, New York, NY, USA).

### 2.7. Antilisterial Activity of EOs

EOs were diluted using Tween 20 (0.5%, *v*/*v*) [45] at the following concentrations 0.5, 0.25, 0.15, and 0.1% (*v*/*v*) and filtered using sterilized nylon syringe filters (0.22 µm). The antimicrobial activity was assessed by a standard disc diffusion assay [46]. Briefly, MHA plates were inoculated using 200 μL of bacterial suspension (10^6^ CFU/mL) on the agar surface. Afterward, the paper disk (6 mm; Biomérieux, Lyon, France) was impregnated with 20 μL of each concentration and placed on MHA plates surface (Oxoid, Badhoevedorp, Netherlands). As a control, Tween 20 was used. The prepared plates were then incubated for 24 h at 37 °C. Ampicillin (10 μg/disk) was used as reference controls. The antibacterial activity was assessed by measuring the mean of inhibitory zones diameters. The experiment was performed in triplicates.

An EO that exhibited efficient antibacterial activity was selected to detect the minimum inhibitory concentration (MIC) and the minimum bactericidal concentration (MBC) by the broth dilution technique. Briefly, one colony of each bacterial strain was sampled and inoculated in 25 mL brain heart infusion broth and incubated at 37 °C for 24 h to obtain a bacterial suspension of 10^9^ CFU/mL, then diluted with buffered peptone water to achieve 10^5^ CFU/mL bacterial suspensions. Serial dilutions of EOs with a concentration ranging from 0.125–2.5% *v*/*v* were prepared with brain heart infusion broth and mixed with bacterial suspensions to give a volume of 4 mL and a final concentration of bacteria of approximately 5 × 10^4^ CFU/mL and incubated at 37 °C for 24 h. The MIC was considered as the lowest concentration that inhibits visible growth. The MBC_90_ was determined by subculturing 100 μL from each negative test tube onto plate count agar plates. MBC_90_ was defined as the lowest concentration of the antimicrobial agent that inhibits ≥ 90% of bacterial isolates.

### 2.8. Detection of Essential Oil Bioactivity

#### 2.8.1. Time–Kill Assay

The effect of EOs on *L. monocytogenes* viability was investigated using a time–kill assay, according to Li et al. [47]. Briefly, a bacterial culture treated with EOs at the MIC was taken as the treatment sample, and dimethyl sulfoxide (5%, DMSO) was used as a control. The prepared cultures were incubated for 8 h at 37 °C. The samples were collected every 2 h and diluted in phosphate-buffer (pH 7.4), then inoculated over the nutrient agar and incubated for 24 h at 37 °C. Bacterial colonies in the treatment and control plates were counted and presented in Log10 (CFU/mL).

#### 2.8.2. Cytoplasmic Membrane Permeability

*L. monocytogenes* cytoplasmic membrane permeability of the selected strains after the treatment using EOs was assessed according to Ye et al. [48] by evaluating the released ions into the bacterial solution supernatant, which increased the relative conductivity. This increase was assessed using a conductivity meter (Shanghai Precision Instruments Co., Ltd., Shanghai, China). The permeability of the cytoplasmic membrane was assessed based on the following equation:

(1) $$\mathrm{Relative}\;\mathrm{conductivity}\;\left(\%\right)=\left(\frac{{\mathrm{RC}}_{2}-{\mathrm{RC}}_{1}}{{\mathrm{RC}}_{0}}\right)\times 100$$

where RC_0_ was the electrical conductivity of dead bacterial cells in glucose (5%) after treatment for 5 min in boiling water; RC_1_ expresses the electrical conductivity of EO at the MIC mixed with glucose (5%); and RC_2_ expresses the electrical conductivity of the treated bacterial culture with EO in the course of 12 h of incubation.

#### 2.8.3. Potassium Ion Leakage Assay

The amount of leaked free potassium ions (K^+^) from the tested *L. monocytogenes* strains was evaluated as reported by Bajpai et al. [49]. After the treatment of *L. monocytogenes* cells with EOs at MIC mixed with 0.1% peptone water and incubated at 37 °C for 8 h, the concentration of extracellular free ions (K^+^ concentration) was tested every 2 h with a Kalium Potassium kit (Quantofix, Macherey-Nagel GmbH & Co. KG, Duren, Germany). *L. monocytogenes* ATCC 19115 was obtained from Food Control Department, Zagazig University, then treated with 5% DMSO and used as a control.

#### 2.8.4. Cell Membrane Integrity Assay

The cell membrane integrity of the selected *L. monocytogenes* strains due to the action of EOs was evaluated as described by Carson et al. [50]. Bacterial samples were treated with the MIC of the most effective EO for each strain, while the control sample was treated with 5% DMSO. All samples were incubated at 37 °C. Samples were obtained every 30 min then centrifuged at 1000× *g* for 10 min. The supernatant absorbance (OD_260_ nm) was measured using a spectrophotometer (SP-3000 plus, Optima, Tokyo, Japan). The absorbance corrections for the treated and control samples were performed by detecting the EOs values without bacteria in sterile peptone water.

#### 2.8.5. Loss of Salt Tolerance Capacity Assay

As a result of EOs treatment, *L. monocytogenes* salt tolerance capacity loss was evaluated as described by Miksusanti et al. [51]. In brief, the treated bacterial culture using EOs at MIC was grown on nutrient agar plates containing various concentrations of NaCl (0, 2.5, 5.0, and 10.0%). Plates treated with 5% DMSO were used as a control. The prepared plates were incubated for 24 h at 37 °C. Bacterial colonies were counted in samples and control plates and presented in Log10 (CFU/mL).

### 2.9. Statistical Analysis

The data obtained were expressed as the means ± standard deviation. One-way analysis of variance was used to detect the significant differences at *p* < 0.05 using GraphPad Prism version 8. Heatmap representations with cluster analysis were performed using NCSS 2021 Statistical Software (NCSS, LLC. Kaysville, UT, USA, ncss.com/software/ncss).

## 3. Results and Discussion

### 3.1. Prevalence and Phenotypic Characterization of L. monocytogenes

As shown in Figure 1, out of 117 bacterial isolates from 75 meat samples, 78 isolates were identified and confirmed as *L. monocytogenes.* Out of 75 tested samples, *L. monocytogenes* were detected in 36/75 (48%) samples. The prevalence of *L. monocytogenes* was 15/36 (41.7%) in the liver, 10/36 (27.8%) in the breast, and 11/36 (30.5%) in the thigh samples.

Our results were in agreement with Elmali et al. [52], who found a 37.5% prevalence of *L. monocytogenes* in 120 retail poultry meat samples. Additionally, Ceylan et al. [53] reported similar results and found that 32.76% prevalence of *L. monocytogenes* was found in 38 of 116 analyzed samples. Escudero-Gilete et al. [54] reported 26% prevalence in the retailed chicken meat samples. However, Goh et al. [55] found that *L. monocytogenes* was detected in 20% of the collected samples, and the prevalence of *L. monocytogenes* was relatively high in the breast meat samples (42.03%) followed by drumstick (11.27%) and thigh meat samples (7.14%). As a result, the high prevalence of *L. monocytogenes* in fresh retail chicken meat, possibly due to poor sanitary quality control, may cause a risk to human health.

### 3.2. L. monocytogenes Drug Resistance and Their Drug Resistance Patterns

In our study, the susceptibility of 78 *L. monocytogenes* isolates to 14 antimicrobial agents is listed in Table 1. The resistance of *L. monocytogenes* isolates against ERY, GEN, and TET was 61.5, 73.1, and 61.5%, respectively. However, 66.7% of *L. monocytogenes* isolates showed susceptibility to AMP and VAN. Moreover, the susceptibility to RIF, CHL, and IPM was 64.1, 60.3, and 60.3%, respectively. The minimum effectiveness was revealed by CIP, CLI, LNZ, NAL, OXA, and TMP that presented susceptibility ranged from 30.8 to 56.4% of all *L. monocytogenes* strains.

The resistance profile of each *L. monocytogenes* isolate was presented in Supplementary Materials, Figure S1. The clustering analysis of these strains was carried out to select the MDR *L. monocytogenes* isolates. Fifty isolates (64.1%) were categorized as MDR *L. monocytogenes*, and their MAR indices ranged from 0.0 to 0.93, with an average of 0.43. The MAR index higher than 0.14 in our study referred to the tested *L. monocytogenes* strains originated from a high-risk source where antimicrobial agents are commonly used [56].

As shown in Table 2, drug resistance patterns (DRPs) obtained from the MDR strains were listed in 18 different DRPs. The most common DRPs were P1, P7, P5, and P5a, which included 18 isolates. However, P1a, P1b, P1c, and P3c included 12 isolates. These eight patterns included 60% (30/50) of all tested MDR strains. As a result, one MDR *L. monocytogenes* strain was chosen randomly from each DRP for further investigation.

Mpondo et al. [57] reported that *Listeria* spp. were found to have phenotypic resistance to erythromycin, sulfonamides, chloramphenicol, aminoglycosides, *β*-lactams, streptomycin, and tetracycline. As a result, antibiotic-resistant Listeria has emerged as a growing One Health concern, exacerbating the worldwide antibiotic resistance crisis and weakening gains in healthcare, food production, and life expectancy, while posing health concerns to the environment, humans, and animals. Olaimat et al. [58] stated that antibiotic resistance of *L. monocytogenes* isolated from food frequently utilized in the treatment of human listeriosis, such as gentamicin, ampicillin, and tetracycline, had been reported. According to Teuber [59], *L. monocytogenes* is susceptible to a wide variety of drugs with antibacterial effects against Gram-positive bacteria, such as ampicillin, gentamicin, erythromycin, and tetracyclines. However, most of the *L. monocytogenes* strains were resistant to cefepime, fosfomycin, cefotaxime, lincosamides, and oxacillin [60]. Additionally, our findings were similar to Bouymajane et al. [6], who found that most *L. monocytogenes* strains originating from different food samples were resistant to tetracycline (20.0%), ampicillin, and sulfamethoxazole/trimethoprim (33.0%), sulfamethoxazole (40.0%), erythromycin (60.0%), and amoxicillin/clavulanic acid (67.0%). They also found that MDR strains represented 66.7% of all isolated strains. Furthermore, vancomycin, rifampicin, and linezolid have been reported as effective agents in listerial infection disease [61]. Additionally, it has been proposed that rifampicin, considered effective against intracellular *L. monocytogenes* and may permeate the cerebrospinal fluid, could aid in the elimination of remaining bacteria [62].

Currently, antibiotic resistance has developed in *L. monocytogenes* isolated from the environment and foods, notably for antibiotics frequently used to treat listeriosis. As a result of the evolution of resistant strains, it is essential to monitor changes in the antibiotic resistance of *L. monocytogenes*. In this context, our results were different than those reported by Chen et al. [63], who found that none of the 72 *L. monocytogenes* strains isolated from the aquatic product was MDR strain. Therefore, we can conclude that the acquired resistance against antimicrobial agents varies based on the bacterial habitat and environment.

### 3.3. Drug Resistance Genes and Virulence Factors

The incidence of genes associated with the drug resistance described was investigated. As a result, the frequency of antibiotic resistance-encoding genes was detected (Figure S2). Out of 18 MDR *L. monocytogenes* selected isolates, the incidence of *erm*B and *aad*A genes was 72% (13/18) and 67% (6/18), respectively. The incidence of *pen*A and *flo*R genes was 61% (11/18) for each gene. However, *cml*A, *ere*A, and *ere*B express 44% (8/18) for each gene, and *amp*C, *van*A, *van*B, *tet*A, and *tet*B incidence was 33% (6/18), 33% (6/18), 39% (7/18), 28% (5/18), and 28% (5/18), respectively. Similar results were reported by Srinivasan et al. [64], who investigated the presence of resistance genes of *L. monocytogenes* isolated from the dairy farms and found a high presence of *flo*R, *pen*A, *tet*A, *str*A, and *sul*I. Additionally, resistance genes for different antibiotic classes are frequently found on the same plasmid. Moreover, as shown in Supplementary Materials, Figure S2, *inl*A, *prf*A, *plc*B, and *acta*A were the most prevalent genes in MDR isolates representing 94.4% (17/18), 88.9% (16/18), 83.3% (15/18), and 83.3% (15/18), respectively. The incidence of resistance genes showed that *hly*A, *plc*A, and *inl*J genes were 77.8% (12/18). However, *fla*A, *lap*, *inl*B, and *inl*C incidence was 72.2% (13/18), 66.7% (12/18), 56.6% (10/18), And 61.1% (11/18), respectively. Van Stelten et al. [65] found that a large proportion of *L. monocytogenes* isolated from food production sources showed a low pathogenicity. However, our results showed that all tested *L. monocytogenes* strains harbor at least 63% of tested virulence genes, which reflect their high pathogenicity. Our results were in agreement with Rugna et al. [66], who found that all investigated *L. monocytogenes* strains had all tested virulence genes. Shen et al. [67] reported that the incidence of *inl*C and *inl*J increases the pathogenicity of *L. monocytogenes* virulence factors. Therefore, our results suggest that *L. monocytogenes* strains originating from food are potentially virulent and could have an essential role in epidemics, regardless of their source.

### 3.4. L. monocytogenes Biofilm-Forming Ability

As shown in Supplementary Material, Figure S2, among all selected L. monocytogenes strains, 88.9% (16/18) have the ability to form biofilm. Of those, 44.4% (8/18) of MDR strains showed strong biofilm formation ability with MPA (OD_600_), ranging from 0.11 to 0.48, while 22.2% (4/18) showed moderate biofilm-forming ability with MPA (OD_600_), ranging from 0.17 to 0.19. However, 33.3% (6/18) showed a weak ability to form a biofilm with MPA (OD_600_) ≤ 0.1.

*L. monocytogenes* can form biofilms as a strategy to survive in food processing environments because cells in biofilms typically exhibit increased tolerance against antimicrobial agents in comparison with their planktonic counterparts [68]. The colonization of *L. monocytogenes* on the surfaces of food processing equipment is enabled by the attachment and production of biofilm [69]. However, the presence of different virulence factor genes such as *prf*A and *hly* is essential for effective biofilm formation by *L. monocytogenes* [70].

In our study, the presence of adhesion protein (*lap*) and internalin proteins (*inlA*, *inlB*, *inlC*, and *inlJ*) was observed in all strong biofilm-forming strains. Similarly, the occurrence of strong BFA in two isolates could be attributed to interruption or overexpression of biofilm-related genes [71].

**Figure 2:**
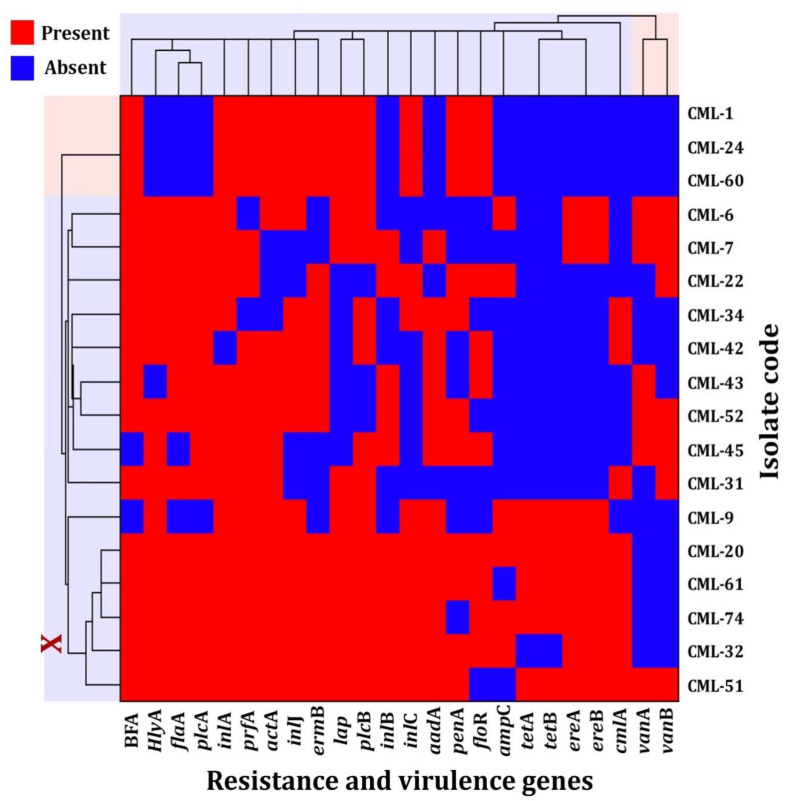
Clustering analysis of resistance and virulence genes-producing MDR *L. monocytogenes* strains: *plc*A, *plc*B, *prf*A, and *act*A, LIPI-1 pathogenicity island; *inl*A, *inl*B, *inl*C, and *inl*J, internalin proteins; *lap*, adhesion protein; *hly*A, listeriolysin O gene, *flaA*, flagellin protein; *aad*A, aminoglycoside adenyltransferase; *amp*C, *β*-lactamase–ampicillin resistance gene; *ere*A, *ere*B, and *erm*B, erythromycin resistance genes; *pen*A, penicillin-binding protein gene; *flo*R, florfenicol export protein; *cml*A, chloramphenicol transporter nonenzymatic chloramphenicol-resistance protein; *tet*A and *tet*B, tetracycline resistance genes; *van*A and *van*B, vanillate o-demethylase oxygenase subunit; BFA, biofilm-forming ability.

**Figure 3:**
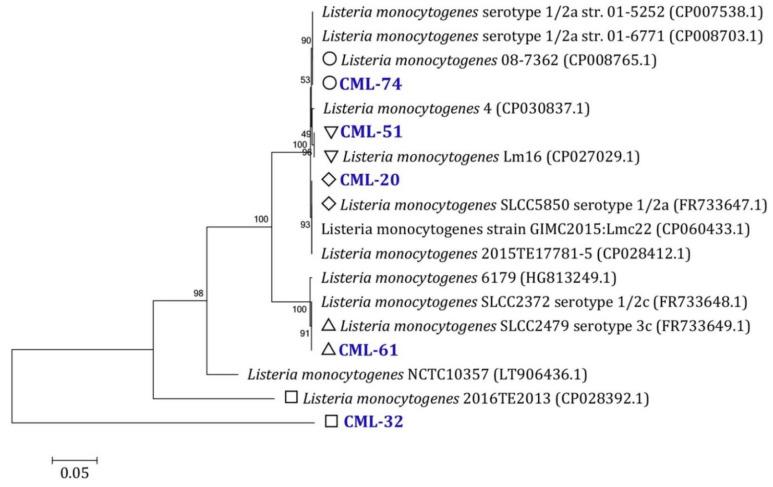
Neighbor-joining tree based on 16S rRNA gene sequences showing the placement of the phylogenetic position of the selected *L. monocytogenes* strains within closely related taxa. Bootstrap value (≥50%) derived from 1000 replicates.

In this study, among all tested MDR *L. monocytogenes* (18 isolates), five isolates had the maximum number of tested resistance and virulence genes. In addition to their ability to form biofilm was arranged into a cluster X, as shown in Figure 2. Identification and creation of a phylogenetic tree of the selected isolates were carried out using 16S rRNA gene sequencing (Figure 3). CML-32, CML-51, CML-61, and CML-74 showed 100% identity to *Listeria monocytogenes* 2016TE2013 (CP028392), *Listeria monocytogenes* Lm16 (CP027029), *Listeria monocytogenes* SLCC2479, serotype 3c (FR733649) and *Listeria monocytogenes* 08-7362 (CP008765), respectively. However, CML-20 showed an identity of 99.78 with *Listeria monocytogenes* SLCC5850 serotype 1/2a (FR733647).

### 3.5. Essential Oil Antilisterial Activity

The chemical composition analysis of the tested EOs are listed in Table 3, showing the main constituent content obtained from a peak area relative to the total peak area in GC–MS analysis. GC–MS analysis showed a high percentage of cinnamaldehyde (63.4%), carvacrol (67.2%), γ-terpinene (22.8%), geranyl acetate (51.7%), linalool (62.2%), linalyl acetate (35.2%), and 1,8-cineole (28.5%) in CBO, TWO, TRO, TGO, CRO, LTO, and RO, respectively.

**Figure 4:**
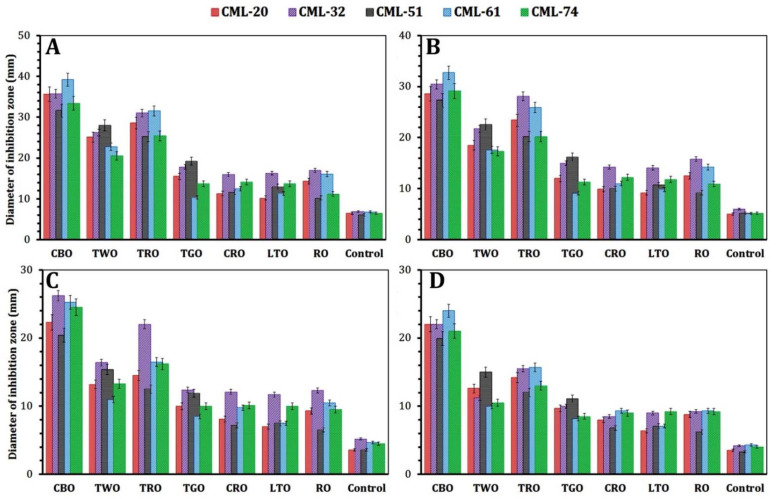
Antilisterial efficiency of different concentrations of essential oils against the selected *L. monocytogenes* strains: inhibitory effect at 0.5% (*v*/*v*) (**A**), 0.25% (*v*/*v*) (**B**), 0.15% (*v*/*v*) (**C**), and 0.1% (*v*/*v*) (**D**).

The antibacterial activity of different EOs was investigated, as shown in Figure 4. Our results showed the efficiency of CBO, TWO, and TRO as antilisterial agents. However, CBO was the most effective antibacterial agent against all resistance and virulence genes-producing MDR *L. monocytogenes*. The concentration of 0.5 and 0.25% showed a significant (*p* < 0.05) inhibitory effect against the selected strains for all tested EOs than 0.15 and 0.1%. At 0.5%, the inhibition zone ranged from 39.2 mm in CBO against CML-61 to 10.2 mm in LTO and RO against CML-20 and CML-51, respectively. At 0.25%, the inhibition zone ranged from 32.7 mm in CBO against CML-61 to 9.1 mm in TGO against CML-61. However, at 0.15%, the inhibition zone ranged from 26.2 mm in CBO against CML-32 to 6.5 mm in RO against CML-51. At 0.1%, the inhibition zone ranged from 24.0 mm in CBO against CML-61 to 6.2 mm in RO against CML-51. Therefore, the CBO, MIC, and MBC_90_ values were detected, and our results showed that the MIC values varied from 0.25 to 0.5% (*v*/*v*) while MBC_90_ values ranged from 0.5 to 1.5% (*v*/*v*).

Our findings were in agreement with Hoque et al. [72], who reported that CBO reduced *L. monocytogenes* in ground chicken meat by 2.0 Log CFU/g in one day. Additionally, Somrani et al. [15] showed that onion, garlic, and cinnamon essential oil were effective towards *L. monocytogenes* and are natural promising antimicrobial alternatives for food manufacture application. Additionally, Hussain et al. [16] stated that 0.025–0.05% of CBO addition was effective by stabilizing color and pH and retarding lipid oxidation through meat storage. The use of 0.5% of CBO leads to stability of color regardless of the bacteria growth reduction compared to other treatments. It can be suggested that adding 0.025% and 0.05% of CBO in meat can be considered as an option to sustain the quality of meat products.

### 3.6. CBO Bioactivity as an Antilisterial Agent

**Figure 5:**
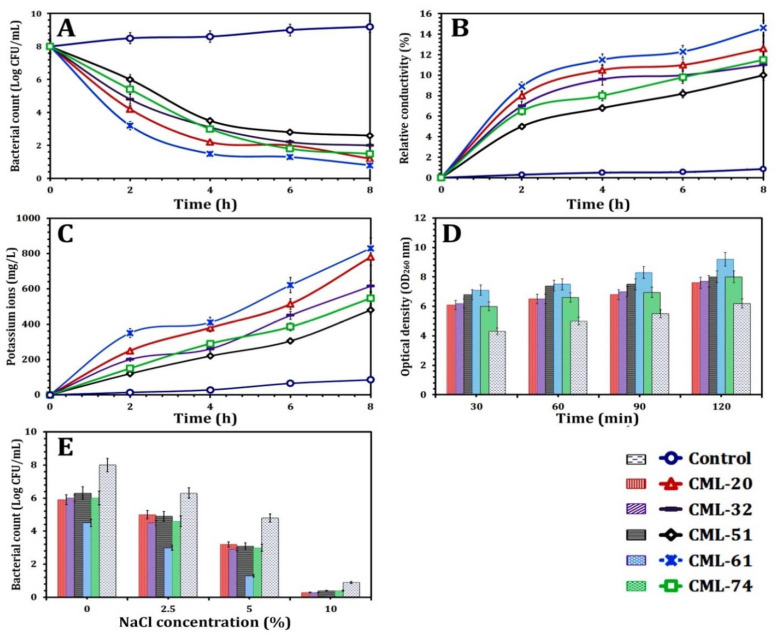
The antilisterial mechanism assays of cinnamon essential oil: (**A**) time–kill assay, (**B**) permeability of cell membrane, (**C**) leakage of potassium ions, (**D**) cell membrane integrity, and (**E**) loss of salt tolerance capacity.

Because of the notable inhibitory effect of CBO, the antilisterial mode of action of CBO against the selected *L. monocytogenes* strains was investigated using different techniques. As shown in Figure 5A, the viability of the bacterial cells decreased along with the incubation time compared to control. Our results also showed that the CML-61 strain was the most inhibited strain after 8 h of incubation due to the action of CBO followed by CML-20, CML-74, CML-32, and CML-51 with a bacterial count of 0.8 ± 0.1, 1.2 ± 0.1, 1.5 ± 0.2, and 2.6 ± 0.4 Log CFU/mL, respectively.

Moreover, the relative electrical conductivity showed a rapid increase for all tested bacterial strains treated with CBO, along with incubation time until the first 4 h. As shown in Figure 5B, CML-61 showed the highest susceptibility to CBO with an increase in the relative conductivity (14.6%) along with incubation time compared to control (0.85%), followed by CML-20 (12.6%), CML-74 (11.5%), CML-32 (11.1%), and CML-51 (10.1%).

Additionally, the loss of K^+^ ions from MDR *L. monocytogenes* tested strains treated with CBO confirms the concept that EOs acts by rupture and disrupts the bacterial cell membrane. After 8 h of incubation, a considerable increase indicated significant leakage of K^+^ ions (Figure 5C). The concentration of K^+^ ion reached 830 ± 55, 780 ± 40, 615 ± 33, 547 ± 44, and 480 ± 25 mg/L in cinnamon treated CML-61, CML-20, CML-51, CML-74 and CML-51, respectively.

Concerning cell membrane integrity assay, the concentration of 260 nm absorbing materials was evaluated, as shown in Figure 5D. For 120 min of incubation, the concentration of 260 nm absorbing compounds in culture filtrates treated with CBO at the MIC concentration gradually increased for all tested strains. CML-61 was the most susceptible strain to CBO and showed a significantly (*p* < 0.05) higher absorbance (OD_260 nm_ = 9.2) than control (OD_260 nm_ = 6.2) and other *L. monocytogenes* strains after 60 min. However, the other tested *L. monocytogenes* strains showed significant (*p* < 0.05) high absorbance than control, and insignificant variation was observed (*p* > 0.05).

Our study also revealed the effect of salt on *L. monocytogenes* cells treated with CBO at the MIC showed a gradual decline in viable cells number compared to the control sample. As shown in Figure 5E, there was insignificant variation (*p* > 0.05) in the bacterial count for CML-20, CML-32, CML-51, and CML-74 at different concentrations of NaCl and significant (*p* < 0.05) decrease in comparison with control. However, CML-61 showed significant decreases in comparison with other strains and control.

**Figure 6:**
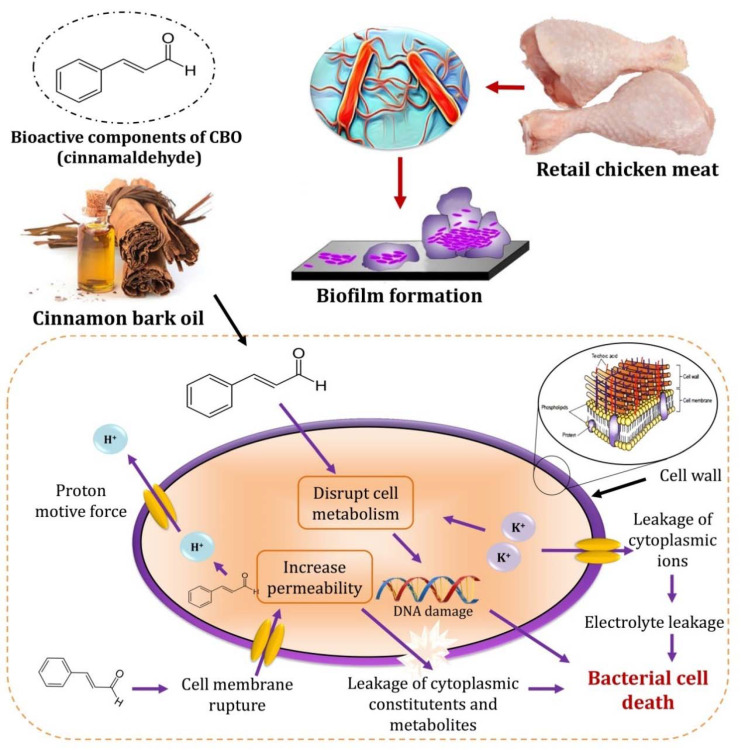
Proposed antibacterial mechanism of cinnamon essential oil against *L. monocytogenes*.

Due to the hydrophobic nature of cinnamaldehyde [13], it has the ability to penetrate the cytosol of treated bacterial cells easily because of the nature of Gram-positive bacterial membrane and leads to the rupture and damage of the cell membrane and consequently leads to cell lysis [73]. However, Cherrat et al. [14] investigated the effect of EOs on the viability of *E. coli* and *Listeria innocua* and reported that Gram-positive bacteria such as *Listeria* spp. are more susceptible to EOs than Gram-negative bacteria due to cell wall hydrophobic nature. Huang et al. [74] reported that CBO is an efficient agent towards foodborne pathogens by inhibiting microbes by various mechanisms such as rupture of the cell wall and the cytoplasmic membrane. Additionally, CBO causes leakage of cellular components, changes in fatty acid and phospholipid constituents, and inhibits protein translocation and genetic materials formation [75]. The significant leakage of cytosolic components was interpreted as an indicator of severe irreversible damage to the cell membrane [76]. Thus, ions and cell metabolites leakage from the CBO-treated bacterial cells could be effective as antilisterial activity. Patra and Baek [73] stated that the selectivity of the bacterial cell membrane plays a significant role in inhibiting the exiting of small ions such as Ca^+2^, Na^+^, and K^+^. This control of membrane permeability is critical to several cellular activities as cell maintenance, transport, and energy transduction. It is thought that cinnamaldehyde could cause abnormalities in the cell membrane, leading to K^+^ leakage and resulting in cellular damage and lysis (Figure 6).

Moreover, the loss of listerial cells’ ability to maintain their growth at a higher salt concentration demonstrates that EOs effects on the bacterial cell membrane led to membrane rupture and decreased the ability of osmoregulation through high salt stress. This impaired ability could potentially impact the membrane’s performance to appropriately regulate the cell under excessive salt concentrations, resulting in cell death [73]. Therefore, our results were in agreement with Zhang et al. [77], who revealed that cinnamon oil caused small electrolyte leakage, resulting in a significant increase in the electric conductivity. Furthermore, the concentration of proteins and nucleic acids in cell suspension was proportional related to cinnamon oil concentration. Additionally, they found that Gram-positive bacteria were more sensitive to cinnamon oil than Gram-negative bacteria. As well, Vasconcelos et al. [78] reported that cinnamon oils and their compounds had been reported to inhibit bacteria by damaging the cell membrane; altering the lipid profile, membrane porins, motility; inhibiting ATPases, cell division, and biofilm formation, and antiquorum sensing effects.

In particular, concerning the food safety applications, Chen et al. [79] reported that cinnamaldehyde is a hydrophobic aromatic aldehyde that has been approved by the FAO/WHO Expert Committee on Food Additives (JECFA) for use as a food flavoring agent. Moreover, Boughendjioua and Djeddi [80] reported that CBO physicochemical and organoleptic properties are very appreciated in perfumery and will be very coveted in the food, cosmetic, and pharmaceutical industry. Furthermore, Goñi et al. [81] and Ju et al. [12] found that CBO has an antimicrobial effect in baked foods without influencing the organoleptic properties. Chitosan films containing CBO has antimicrobial activity, keeping the natural organoleptic characteristics and extending the shelf life [82]. Generally, our results indicate the efficiency of the antilisterial activity of cinnamaldehyde toward resistance and virulence genes-producing MDR *L. monocytogenes* strains, maintaining good nutritional and organoleptic properties of food products.

## 4. Conclusions

*Listeria monocytogenes* is one of the most severe foodborne pathogens. Our results revealed that the prevalence of *L. monocytogenes* in raw chicken meat in Zagazig city was 48%. Seventy-eight isolates were identified as *L. monocytogenes*. Of those, 64.1% of the identified isolates were multidrug resistance bacteria. Moreover, the study of resistance genes on the selected isolate showed that *erm*B, *aad*A, *pen*A, and *flo*R genes were the most prevalent genes. However, the most prevalent virulence genes were *inl*A, *prf*A, *plc*B, and *acta*A genes. On the other hand, the activity of seven different essential oils against MDR *L. monocytogenes* was investigated. Cinnamon bark oil showed high efficiency as an antilisterial agent against MDR *L. monocytogenes* isolated from retail chicken meat. Our results also interpret cinnamon bark oil bioactivity by disrupting the cell membrane leading to a loss of membrane permeability, leakage of the cytoplasm constituents, ions, and metabolites. Therefore, this study could assist in detecting one of the promising antilisterial agents from natural sources that inhibits resistance and virulence genes-producing *L. monocytogenes*, which would improve food safety applications.

## Figures and Tables

**Table 1:** The resistance of *L. monocytogenes* strains against different antimicrobial agents (*n* = 78).

Antibiotics	Concentration (μg/Disc)	Resistant		Intermediate		Susceptible	
		No.	(%)	No.	(%)	No.	(%)
Ampicillin (AMP)	10	22	28.2	4	5.13	52	66.67
Chloramphenicol (CHL)	30	29	37.2	2	2.56	47	60.26
Ciprofloxacin (CIP)	5	29	37.2	5	6.41	44	56.41
Clindamycin (CLI)	2	36	46.2	1	1.28	41	52.56
Erythromycin (ERY)	15	48	61.5	5	6.41	25	32.05
Gentamicin (GEN)	120	57	73.1	7	8.97	14	17.95
Imipenem (IPM)	10	29	37.2	2	2.56	47	60.26
Linezolid (LNZ)	30	29	37.2	5	6.41	44	56.41
Nalidixic acid (NAL)	30	29	37.2	5	6.41	44	56.41
Oxacillin (OXA)	1	29	37.2	4	5.13	45	57.69
Rifampicin (RIF)	5	22	28.2	6	7.69	50	64.10
Tetracycline (TET)	30	48	61.5	6	7.69	24	30.77
Trimethoprim (TMP)	5	36	46.2	5	6.41	37	47.44
Vancomycin (VAN)	30	22	28.2	4	5.13	52	66.67

**Table 2:** The antimicrobial resistance patterns of MDR *L. monocytogens* strains (*n* = 50).

Pattern Code.	Antimicrobial Resistance Pattern	MAR Index	Strains No.
P1	W, X, Y, AMP, OXA, TET, TMP	0.93	5
P1a	X, Y, Z, AMP, LNZ, NAL, OXA	0.93	3
P1b	W, X, Y, Z, AMP	0.93	3
P1c	W, X, Y, Z, OXA	0.93	3
P2	X, Y, LNZ, NAL, TET	0.64	2
P3	AMP, CHL, CLI, IPM, NAL, RIF, TET, VAN	0.57	2
P3a	Y, Z, AMP, CLI	0.57	1
P3b	X, GEN, OXA, RIF, TET, TMP	0.57	2
P3c	W, Y, CHL, CIP	0.57	3
P4	AMP, CIP, IPM, OXA, RIF, TMP, VAN	0.50	2
P4a	Z, AMP, CLI, Y, NAL	0.50	2
P4b	AMP, CLI, IPM, NAL, RIF, TET, VAN	0.50	2
P4c	CIP, CLI, ERY, GEN, LNZ, TET, VAN	0.50	2
P5	CHL, ERY, GEN, NAL, OXA, TET	0.43	4
P5a	ERY, GEN, LNZ, OXA, TET, TMP	0.43	4
P6	ERY, GEN, LNZ, TET, TMP	0.36	3
P7	GEN, OXA, TET, TMP	0.29	5
P8	CHL, ERY, GEN	0.2	2

W: NAL, LNZ, RIF; X: CHL, CIP, CLI; Y: ERY, GEN, IPM; Z: TET, TMP, VAN.

**Table 3:** Essential oils used and their major constituents.

No.	Essential Oil	Code	Main Constituent *	RT	Content (%)
1	Cinnamon bark oil	CBO	Cinnamaldehyde	29.79	63.4
			Cinnamyl acetate	21.73	15.2
2	Thyme (wild) oil	TWO	Carvacrol	18.09	67.2
3	Thyme (red) oil	TRO	Thymol	21.02	17.4
			γ-terpinene	10.11	22.8
4	Thyme (geraniol) oil	TGO	Geraniol	12.45	28.6
			Geranyl acetate	16.33	51.7
5	Coriander oil	CRO	Linalool	25.25	62.2
6	Lavender (true) oil	LTO	Linalool	14.24	31.4
			Linalyl acetate	17.41	35.2
7	Rosemary oil	RO	1,8-cineole	12.43	28.5
			α-pinene	7.07	19.3
			Camphor	18.01	16.2

* The main constituent content was detected from a peak area relative to the total peak area in GC–MS analysis. The main constituents content higher than 15% are listed.

## Data Availability

The datasets generated and/or analyzed during the current study areavailable from the corresponding author on reasonable request.

## Supplementary Materials

The following are available online at https://www.mdpi.com/article/10.3390/foods10123006/s1, Figure S1: Clustering analysis for selecting the MDR *L. monocytogenes* strains., Figure S2. Incidence of resistance and virulence gene in MDR *L. monocytogenes* strains. Table S1: Antimicrobial agents, zone and interpretive chart., Table S2. PCR Primers and references for 16S rRNA of *L. monocytogens* and virulence genes., Table S3. PCR Primers and references for 16S rRNA of Listeria and virulence genes.
