# Supplementary material for: Bioactivity of Essential Oils for Mitigation of Listeria monocytogenes Isolated from Fresh Retail Chicken Meat
Source: Foods. 2021 Dec 4;10(12):3006. doi: 10.3390/foods10123006 (PMC8701900; doi:10.3390/foods10123006)
Supplement: Supplementary file 1 [file foods-10-03006-s001.zip › foods-1434799-supplementary.pdf]

Article

Bioactivity of Essential Oils for Mitigation of *Listeria monocytogenes* Isolated from Fresh Retail Chicken Meat

Alaa Eldin M. A. Morshdy [1], Mohammed S. Al-Mogbel [2], Mohamed E. M. Mohamed [3], Mohamed Tharwat Elabbasy [1,4], Azza K. Elshafee [1,*] and Mohamed A. Hussein [1]

[1] Food Control Department, Faculty of Veterinary Medicine, Zagazig University, Zagazig 44519, Egypt; A.Morshedy@zu.edu.eg (A.E.M.A.M.); mt.elabbasy@uoh.edu.sa (M.T.E.); elged2010@yahoo.com (M.A.H.)

[2] Clinical Laboratory Sciences Department, College of Applied Medical Sciences, Ha'il University,Ha'il P.O. Box 2240, Saudi Arabia; msm_hhscb@hotmail.com

[3] Zoonoses Department, Faculty of Veterinary Medicine, Zagazig University, 44519, Egypt; bishet68@yahoo.de

[4] Public Health Department, College of Public Health and Health Informatics, Ha'il University, Ha'il 2440, Saudi Arabia

* Correspondence: aza.khaled@vet.zu.edu.eg; Tel.: +2-01147560577

Keywords: *Listeria monocytogenes*, Antimicrobial agents, Essential oils, Multidrug-resistance, foodborne disease, Biofilm.

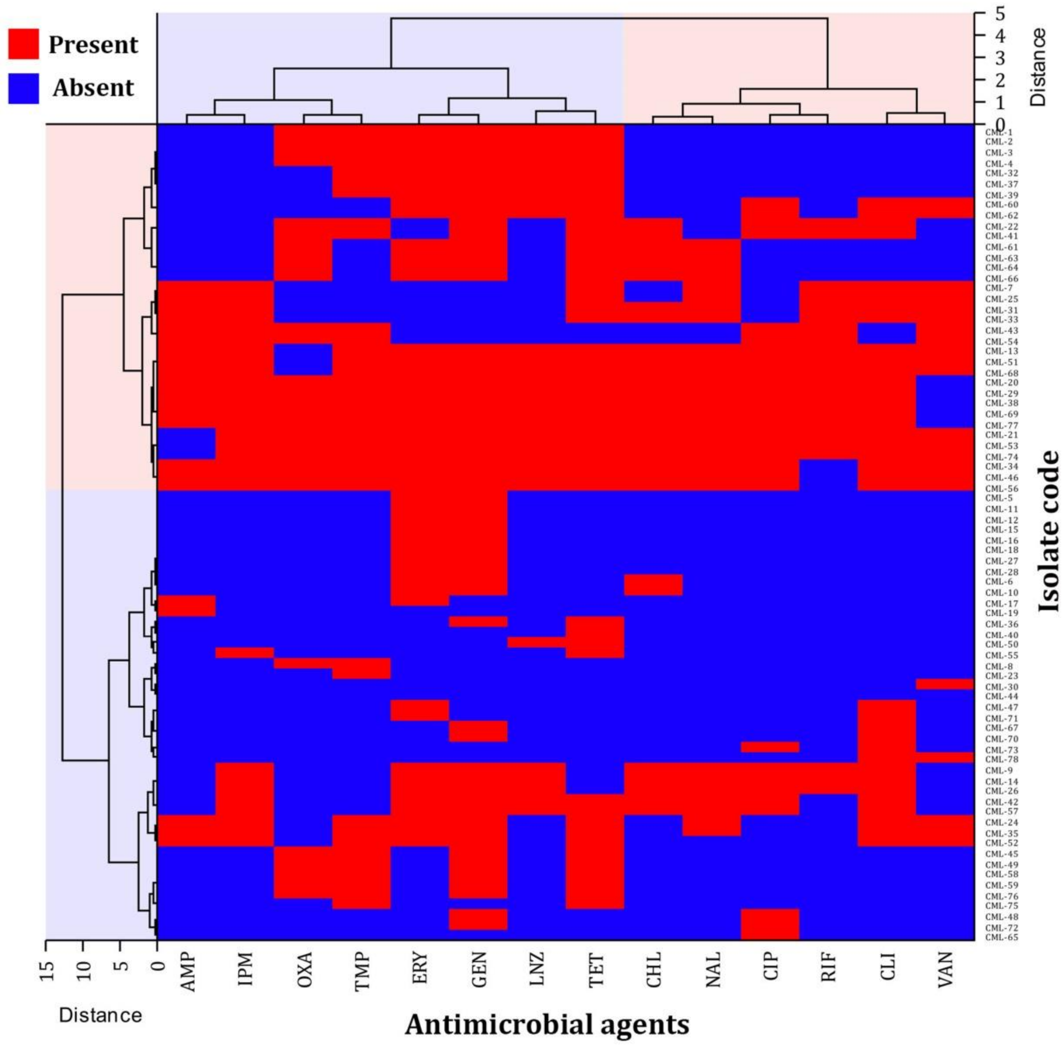

Figure S1. Clustering analysis for selecting the MDR *L. monocytogenes* strains.

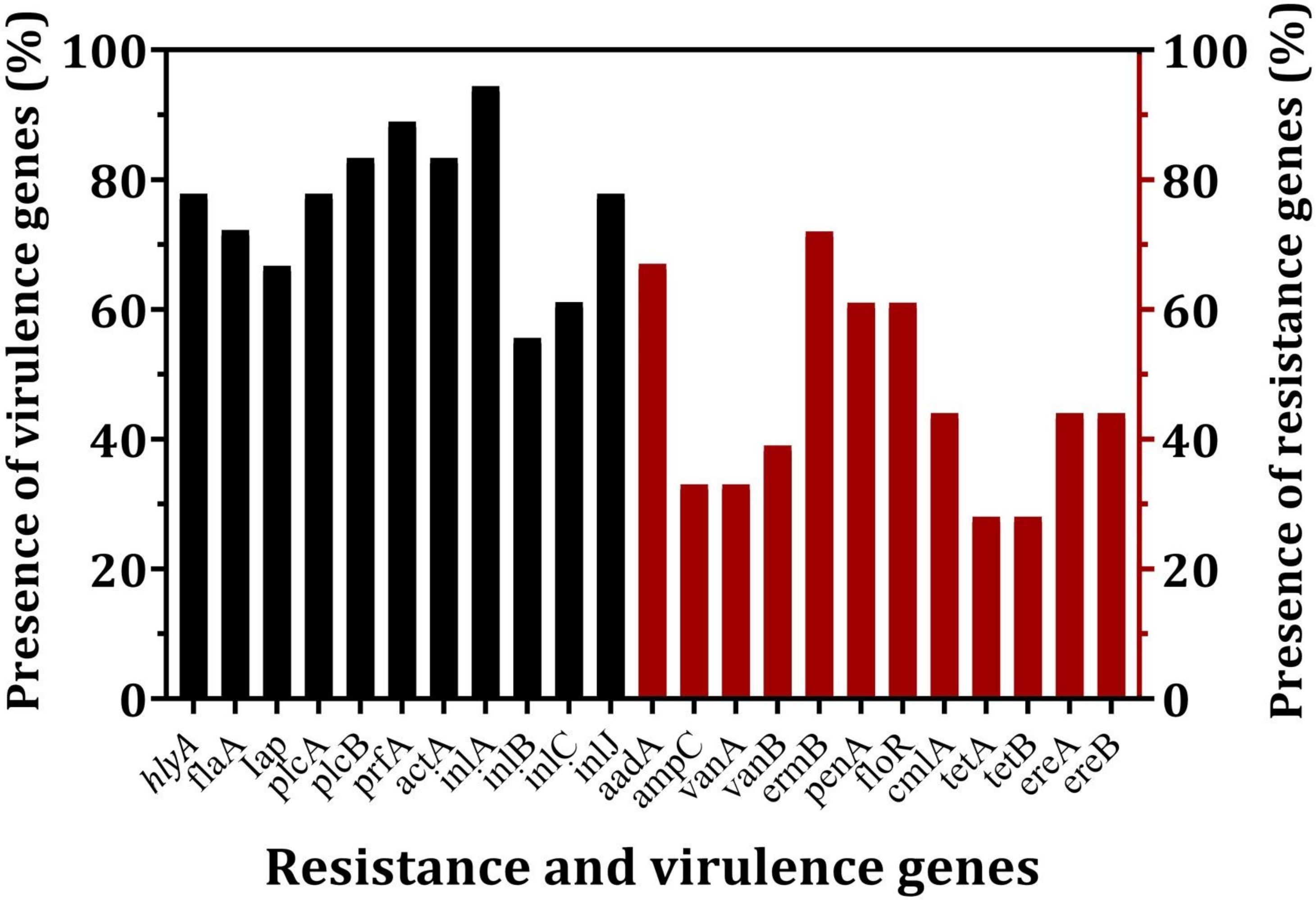

Figure S2. Incidence of resistance and virulence gene in MDR *L. monocytogenes* strains.

Table S1. Antimicrobial agents, zone and interpretive chart.

Class	Antibiotics	Abbreviation	Concentration (µg/disc)	Interpretive categories and zone diameter breakpoints (mm)		
				S	I	R
**Penicillins**	Ampicillin	AMP	10	≥17	14-16	≤13
**Phenicols**	Chloramphenicol	CHL	30	≥ 18	13-17	≤ 12
**Fluoroquinolone**	Ciprofloxacin	CIP	5	≥21	16-20	≤15
**Lincosamides**	Clindamycin	CLI	2	≥ 21	15-20	≤ 14
**Macrolides**	Erythromycin	ERY	15	≥ 23	14-22	≤ 13
**Aminoglycosides**	Gentamicin	GEN	120	≥ 15	13-14	≤ 12
**Carbapenems**	Imipenem	IPM	10	≥ 19	16-18	≤ 15
**oxazolidinones**	Linezolid	LNZ	30	≥ 21	-	≤ 20
**Quinolone**	Nalidixic acid	NAL	30	≥19	14-18	≤13
**B-lactam**	Oxacillin	OXA	1	≥18	-	≤17
**Ansamycins**	Rifampicin	RIF	5	≥ 20	17-19	≤ 16
**Tetracyclines**	Tetracycline	TET	30	≥ 19	15-18	≤ 14
**Folate pathway inhibitors Iclaprim**	Trimethoprim	TMP	5	≥16	11-15	≤10
**Glycopeptides**	Vancomycin	VAN	30	≥17	15-16	≤14

Table S2. PCR Primers and references for 16S rRNA of *L. monocytogens* and virulence genes.

Target gene		Sequence (5'-3')	Amplified product (bp)	Reference
	16S rRNA	F: GGACCGGGGCTAATACCGAATGATAA<br>R: TTCATGTAGGCGAGTTGCAGCCTA	**1200**	28
Listeriolysin O	*Hly*A	F: CCT AAG ACG CCA ATC GAA<br>R: AAG CGC TTG CAA CTG CTC	**702**	29
Flagellin protein	*fla*A	F: AGCTCTTAGCTCCATGAGTT<br>R: AGTAGCAGCACCTGTAGCAGT	**420**	30
Adhesion protein	*Iap*	F: ACAAGCTGCACCTGTTGCAG<br>R: TGACAGCGTGTGTAGTAGCA	**131**	31
LIPI-1 pathogenicity island	*plc*A	F: CTGCTTGAGCGTTCATGTCTCATCCCCC<br>R: ATGGGTTTCACTCTCCTTCTAC	**1484**	32
	*plc*B	F: GCAAGTGTTCTAGTCTTTCCGG<br>R: ACCTGCCAAAGTTTGCTGTGA	**795**	33
	*prf*A	F: CCCCAAGTAGCAGGACATGCTAA<br>R: GTATCACAAAGCTCACGAG	**571**	
	*act*A	F: CGCCGCGGAAATTAAAAAAAGA<br>R: ACGAAGGAACCGGGCTGCTAG	**839**	34
Internalin proteins	*inl*A	F: ATTATGCCAAGTGGTTACGGA<br>R: ATCTGTTTGCGAGACCGTGTC	**800**	35
	*inl*B	F: CATGGGAGAGTAACCCAACC<br>R: GCGGTAACCCCTTTGTCATA	**500**	35
	*inl*C	F: CCCACAATCAAATAAGTGACCTT<br>R: CTGGGTCTTTGACAGTATTTGTT	**400**	
	*inl*J	F: TGTAACCCCGCTTACACAGTT<br>R: AGCGGCTTGGCAGTCTAATA	**238**	35

Table S3. PCR Primers and references for 16S rRNA of Listeria and virulence genes.

Target gene	Sequence (5'-3')	Amplified product (bp)	Reference
*aad*A	F: GTGGATGGCGGCCTGAAGCC<br>R: AATGCCCAGTCGGCAGCG	**525**	37
*amp*C	F: TTCTATCAAMACTGGCARCC<br>R: CCYTTTTATGTACCCAYGA	**550**	
*van*A	F: CATGACGTATCGGTAAAATC<br>R: ACCGGGCAGRGTATTGAC	**885**	
*van*B	F: CATGATGTGTCGGTAAAATC<br>R: ACCGGGCAGRGTATTGAC	**882**	
*erm*B	F: GAAAAGGTACTCAACCAAATA<br>R: AGTAACGGTACTTAAATTGTTTAC	**639**	38
*pen*A	F: ATCGAACAGGCGACGATGTC<br>R: GATTAAGACGGTGTTTTACGG	**500**	39
*flo*R	F: TATCTCCCTGTCGTTTCCAG<br>R: AGAACTCGCCGATCAATG	**399**	40
*cml*A	F: CCGCCACGGTGTTGTTGTTATC<br>R: CACCTTGCCTGCCCATCATTAG	**698**	41
*tet*A	F: GGCCTCAATTTCCTGACG<br>R: AAGCAGGATGTAGCCTGTGC	**372**	42
*tet*B	F: GAGACGCAATCGAATTCGG<br>R: TTTAGTGGCTATTCTTCCTGCC	**228**	
ereA	F: AACACCCTGAACCCAAGGGACG<br>R: CTTCACATCCGGATTCGCTCGA	**420**	43
*ere*B	F: AGAAATGGAGGTTCATACTTACCA<br>R: CATATAATCATCACCAATGGCA	**546**	
